# De Winter electrocardiogram pattern due to type A aortic dissection: a case report

**DOI:** 10.1186/s12872-022-02596-8

**Published:** 2022-04-05

**Authors:** Qing Zhang, Dong-dong Yang, Yi-fei Xu, Yuan-gang Qiu, Zhuo-yi Zhang

**Affiliations:** 1 Department of Neurology, The First Affiliated Hospital of Zhejiang Chinese Medical University (Zhejiang Provincial Hospital of Traditional Chinese Medicine), No. 54 Youdian Road, Shangcheng District, Hangzhou, 310006 Zhejiang China; 2 Department of Emergency Medicine, The First Affiliated Hospital of Zhejiang Chinese Medical University (Zhejiang Provincial Hospital of Traditional Chinese Medicine), No. 54 Youdian Road, Shangcheng District, Hangzhou, 310006 Zhejiang China; 3 Department of Cardiology, The First Affiliated Hospital of Zhejiang Chinese Medical University (Zhejiang Provincial Hospital of Traditional Chinese Medicine), No. 54 Youdian Road, Shangcheng District, Hangzhou, 310006 Zhejiang China

**Keywords:** Aortic dissection, De Winter electrocardiogram pattern, Acute myocardial infarction, Case report

## Abstract

**Background:**

De Winter electrocardiograph (ECG) pattern is an atypical presentation of acute myocardial infarction (AMI) due to severe stenosis of the left anterior descending (LAD). Complications of acute aortic dissection (AD) in the setting of acute myocardial infarction (AMI) with de Winter sign are relatively rare and physicians may easily miss the diagnosis of AD. We report a case of patient with acute chest pain and de Winter ECG pattern due to AD involving the left main coronary artery (LM), LAD and left circumflex artery (LCX).

**Case presentation:**

A 57-year-old male patient was initially diagnosed with AMI and then the diagnosis of acute AD was supported by transthoracic echocardiograph (TTE). After two stents were implanted respectively into the proximal LM-LAD and LM-LCX, he recovered from cardiogenic shock. Two months later, the patient underwent the surgery of ascending aorta replacement. After the surgery, there was no obvious chest discomfort during follow-up.

**Conclusions:**

When an ECG shows a “de Winter pattern”, we should also consider the possibility of AD which result in LAD occlusion. TTE is a useful tool in screening for AD. Further research is needed to prove that percutaneous coronary intervention (PCI) may be a useful treatment strategy in the case of AD leading to severe LAD occlusion and unstable hemodynamics when there’s no condition to perform aortic replacement surgery immediately.

**Supplementary Information:**

The online version contains supplementary material available at 10.1186/s12872-022-02596-8.

## Background

De Winter electrocardiograph (ECG) pattern is estimated to be present in about 2% of patients with acute occlusion of the proximal left anterior descending (LAD) [1]. The pattern consisting of ST-segment depression and hyper acute T waves is considered as ST-segment elevation myocardial infarction (STEMI) equivalent and also requires emergency percutaneous coronary intervention (PCI) [2, 3].

Complications of aortic dissection (AD) in the setting of acute myocardial infarction (AMI) with de Winter sign are relatively rare and physicians may easily miss the diagnosis of AD.To the best of our knowledge, no prior description of the “de Winter pattern” associated with AD has been reported.

## Case presentation

A 57-year-old man was transferred to the Emergency Department of the First Affiliated Hospital of Zhejiang Chinese Medicine University, because of sudden onset severe chest pain for 40 min. The pain was characterized as pressure-like, progressive in nature with radiation to the back region, with profuse perspiration and blurred vision. The patient had been diagnosed with hypertension for 3 years and on losartan 100 mg per day without monitoring blood pressure. Apart from hypertension, his past medical history was clinically unremarkable. He was not on other regular medications before presentation and had no significant family history. He was a current smoker with a 20-year-history of smoking 20 cigarettes per day and drank 100 g per day for 20 years.

On admission, his body temperature was 37.1 °C; pulse 81 beats/min; respiratory rate 20 breaths/min with normal oxygen saturation; and blood pressure 92/61 mmHg.

**Fig. 1:**
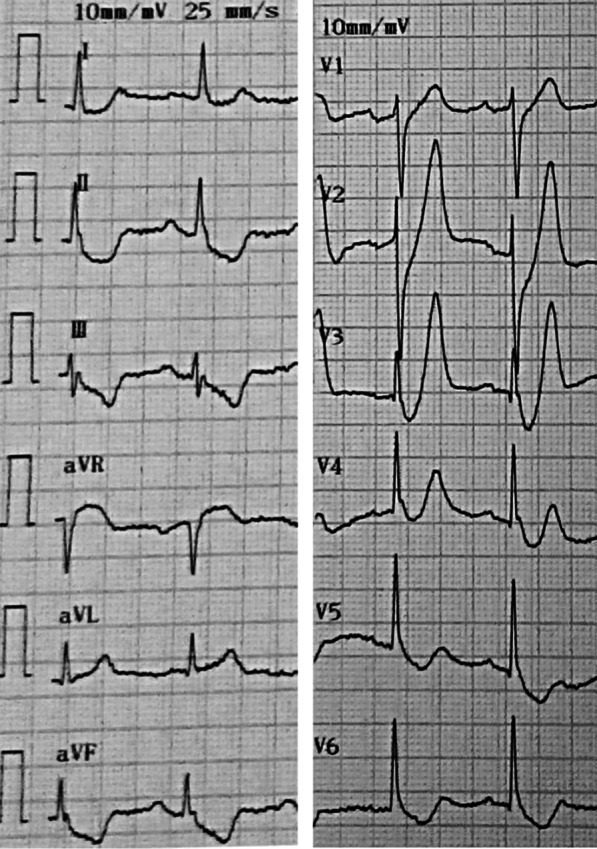
Emergency ECG

Point-of-care testing showed: white blood cell count at 7.1 * 10^9^/L (3.5–9.5 * 10^9^/L), lymphocyte% at 35.8% (20–50%), platelet count at 106 * 10^9^/L (125–350 * 10^9^/L), serum potassium at 3.92 mmol/L (reference value 3.5-5.3 mmol/L), blood creatinine at 75 μmol/L (59–104 μmol/L), creatinine kinase—myocardial band at 15.5 U/L (reference value < 25 U/L), brain natriuretic peptide at 65.8 pg/mL (reference value < 100 pg/mL), cardiac troponin I at 0.003ug/L ( reference value < 0.026 μg/L), and D-dimer at 4.52 mg/L (reference value < 0.55 mg/L). The 12-lead ECG obtained at admission showed ST-segment depression (> 1 mm) at the J point, with tall, symmetrical T-waves in the leads V1–V3 along with ST-segment elevation (1 mm) in the lead a VR (Fig. 1). These ECG changes suggested de Winter syndrome, a condition associated with acute occlusion of LAD.

On the basis of chest pain and de Winter ECG changes, the patient was initially diagnosed with AMI and treated with aspirin 300 mg (chewable), ticagrelor 600 mg (chewable) and atorvastatin 20 mg according to the current guidelines. Emergency coronary angiography (CAG) was immediately performed.

**Fig. 2:**
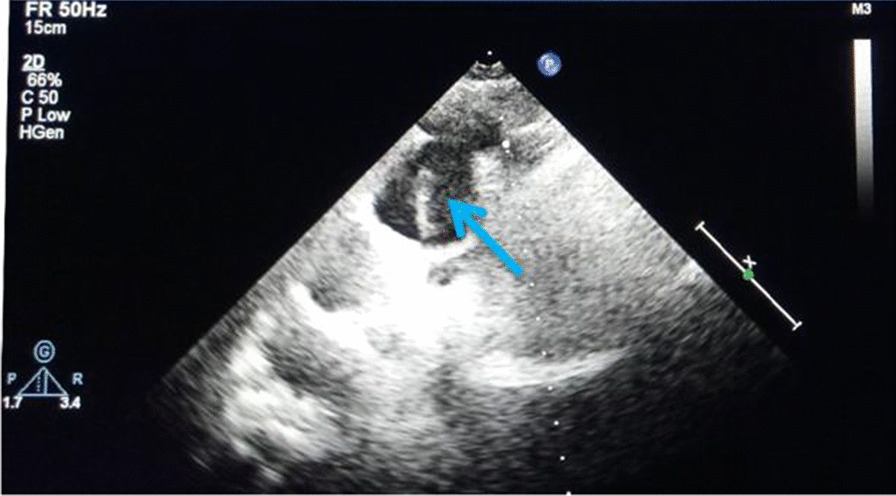
Echocardiographic examination in suprasternal notch long-axis view

**Fig. 3:**
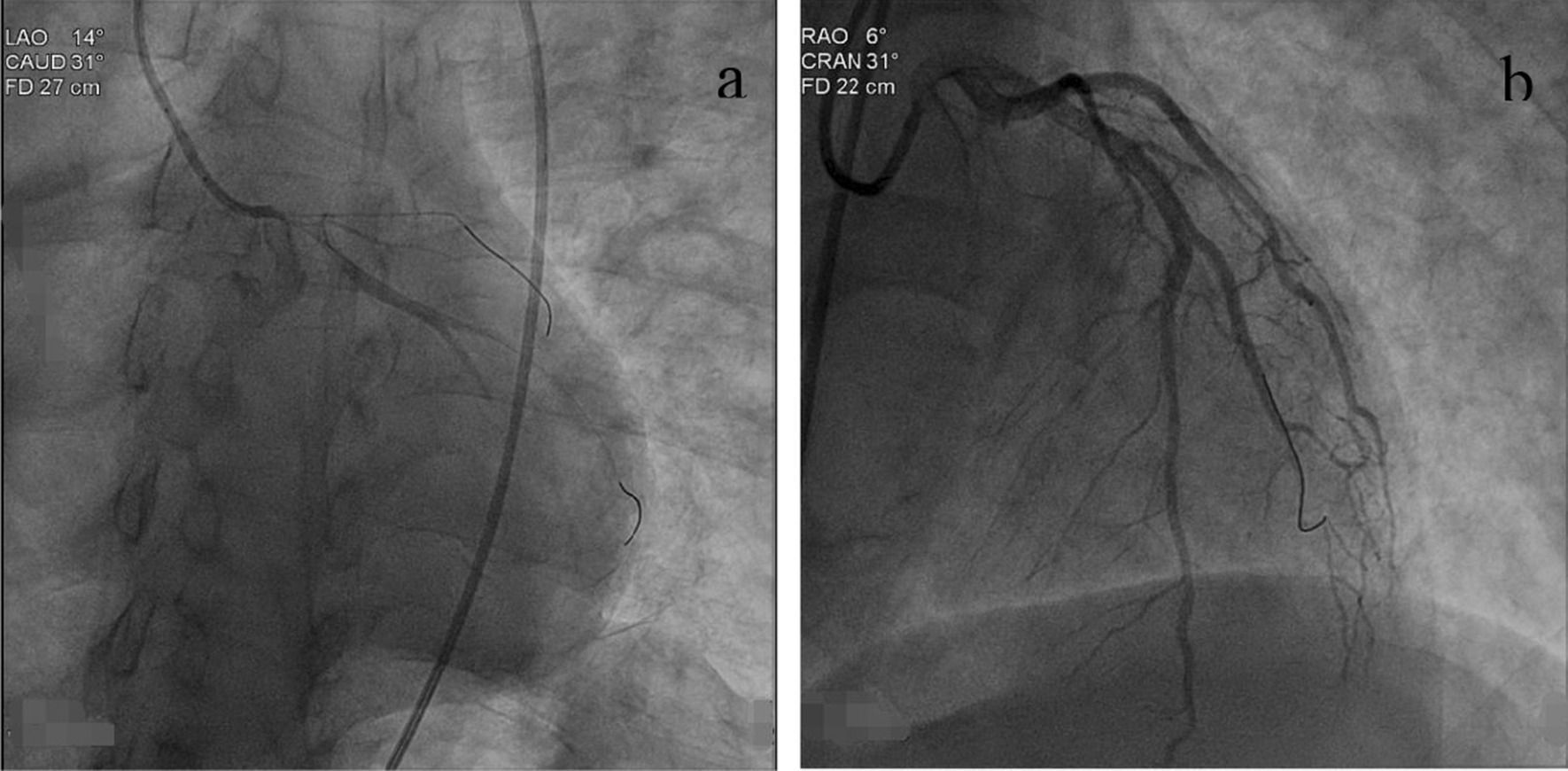
Angiography results before (**a**) and after (**b**) stent implantation

In the catheterization laboratory, the right radial artery was cannulated with a 6-French sheath. The possibility of an AD was indicated by unsuccessful attempt to selectively engage the coronary ostium. Subsequent intraoperative ultrasound showed intimal tear in the aortic arch (Fig. 2), supporting the diagnosis of AD. CAG via femoral artery showed subtotal left main coronary artery (LM) occlusion, total LAD occlusion and a 99% stenosis of the left circumflex artery (LCX) (Fig. 3a; Additional file 1: Video of coronary angiography). The blood pressure of the patient was at 85/56 mmHg with continuous norepinephrine administration, which signaled a poor prognosis.

Considering the hemodynamic instability and high risk of a referral for an emergency aortic computed tomography in CT room and repair surgery in senior surgical hospital, cardiologists advised the patient to undergo PCI urgently. In order to promote rapid recovery from the cardiogenic shock, two 3.5 × 32 mm Promus Element Plus stents were implanted respectively into the proximal of LM-LAD and LM-LCX after the agreement of family members. Following treatment by direct stenting, the LM-LAD and LCX blood flow was completely restored (Fig. 3b; Additional file 1: Video of coronary angiography). Following PCI, the patient was stabilized and shortly admitted to the Intensive Care Unit.

**Fig. 4:**
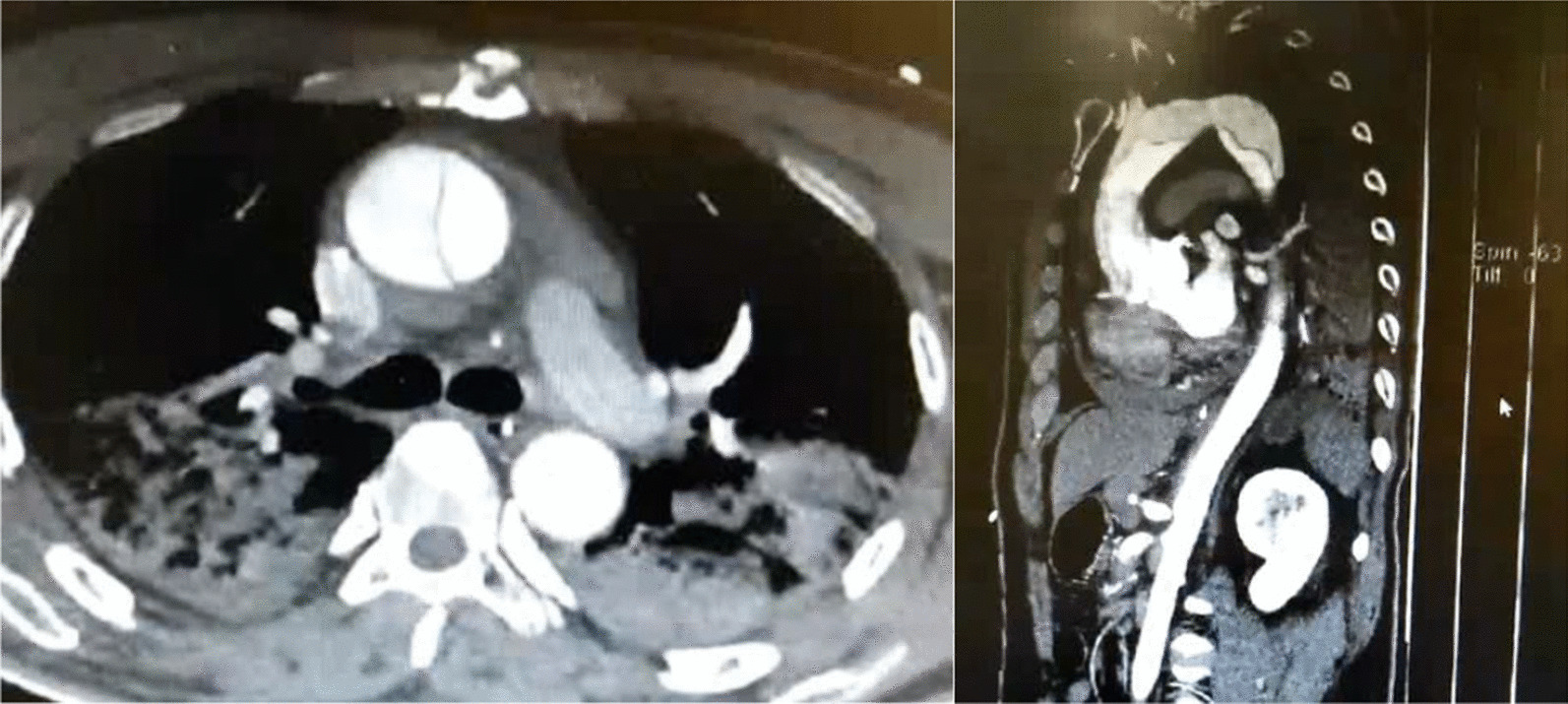
Computed tomography scan of the chest

Aortic computed tomography (CT) angiography confirmed final diagnosis of type A aortic dissection (TAAD) (Fig. 4). The patient still suffered from chest congestion after stabilizing blood pressure and heart rate.

**Fig. 5:**
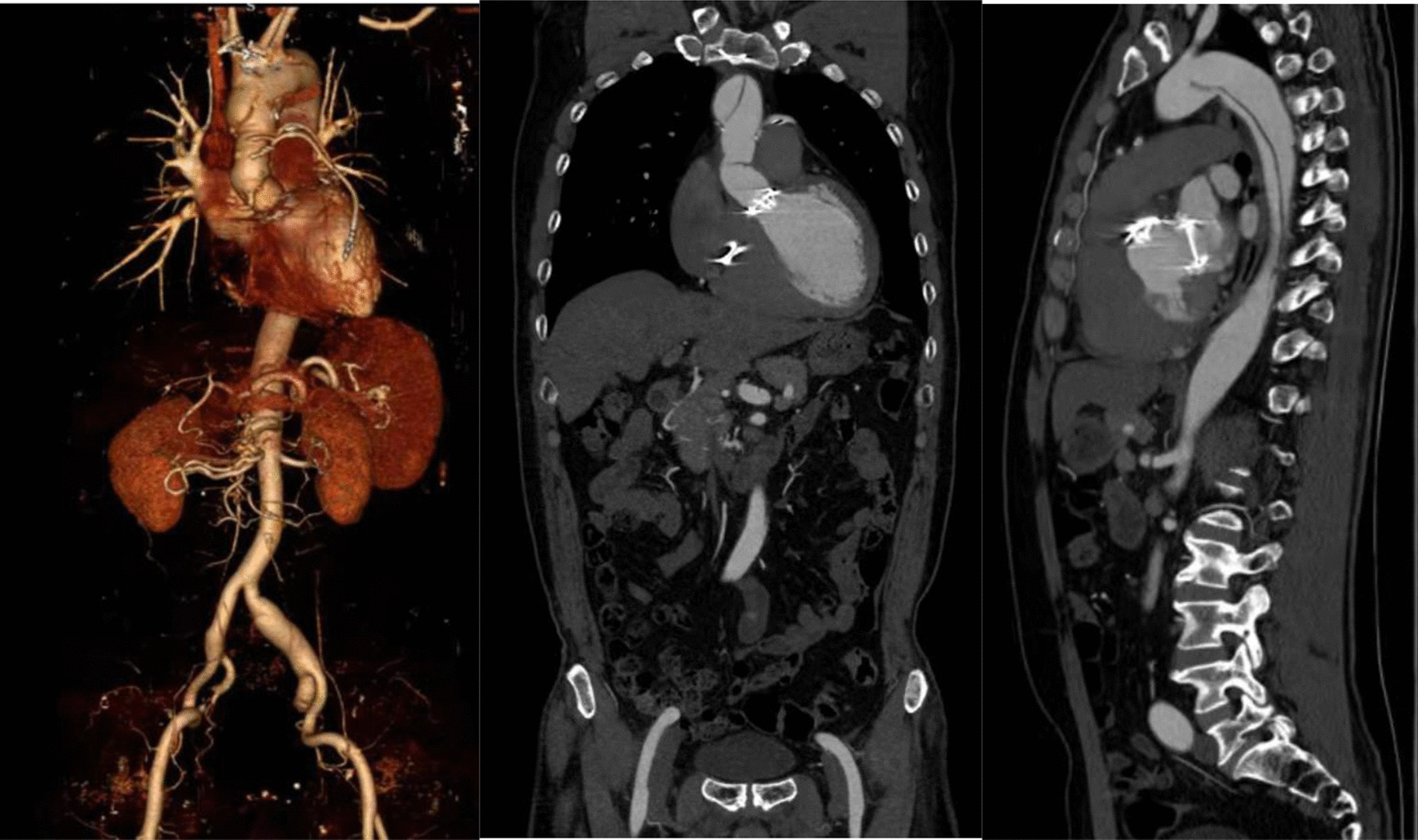
A repeat CT scan of the aorta at the 22ed month after Bentall surgery

Two months after the PCI procedure, transthoracic echocardiograph (TTE) was performed for follow-up, which revealed dilation of the aortic root pseudo cavity, severe aortic regurgitation, severe mitral and tricuspid regurgitation, and pulmonary hypertension. The patient still suffered from post-exercise chest tightness and underwent the Bentall procedure. After the surgery, there was no obvious chest discomfort during follow-up. On the patient’s most recent clinical follow-up visit, a repeat CT scan of the aorta showed replacement of ascending aorta, aortic valve, mitral valve and tricuspid valve, and coronary artery bypass grafting (Fig. 5).

## Discussion and conclusions

De Winter et al. firstly described the new ECG pattern (a 1- to 3-mm upsloping ST-segment depression at the J point followed by peaked, positive T waves in precordial leads; normal or only slightly widened QRS complexes; loss of R wave progression and a 1- to 2-mm ST-elevation in lead a VR) in 2008 [1]. It usually signifies AMI due to LAD occlusion [1], which may be an early ECG pattern in the development of AMI [4, 5]. Therefore, de Winter syndrome should be treated as STEMI equivalent with urgent reperfusion treatment [3]. This patient was initially diagnosed as AMI according to the symptom of chest pain and de Winter ECG.

However, as a result of similar clinical symptoms and ECG changes, acute TAAD can mimic STEMI [6] and non-ST-segment elevation myocardial infarction (NSTEMI) [7]. When a dissecting hematoma or disrupted inner layers of the aortic wall obstruct the orifice of coronary arteries, AD can cause AMI, which are relatively rare with a reported incidence of 7% [8].Because of the fact that dissection more commonly originates from the right anterior aspect of the ascending aorta above the right coronary sinus, the right coronary artery is more often involved in the patients with TAAD [9]. The LCA can also be compressed. Liu et al. found that LAD and LCX were more often involved in patients with type B AD [10]. Myocardial ischemia was classified into 3 types according to the mechanism of coronary artery obstruction due to retrograde AD [11]. Ostial dissection (type A) may not be associated with coronary ischemia unless a local flap is created and obstructs coronary flow with a trapdoor mechanism. In type B (dissection with a coronary false channel), coronary flow impairment results from the compression in diastole of the true lumen by the obstructing false channel. In the third type (circumferential detachment with an inner cylinder intussusception), the dissection may extend along the course of the coronary artery and lead to direct obstruction of coronary blood flow. In this case, de Winter ECG changes might be secondary to the compression of the LM, LAD and LCX by the dissected intimal flap, which was different from the common de Winter syndrome caused by severe stenosis of LAD. As far as we know, this case is the first presentation of de Winter pattern due to AD.

It deserves to be mentioned that de Winter ECG pattern can be observed in patients with spontaneous coronary artery dissection (SCAD) [12], which is defined as a hematoma and/or tear within the coronary arterial wall not due to AD, trauma, atherosclerosis, or iatrogenic injury. LAD is the most common artery affected in SCAD [13] and coronary ischemia is secondary to coronary artery obstruction from intimal disruption. As rare cause of AMI, suspicion for SCAD should be raised in younger women and patients without conventional atherosclerotic risk factors. With the risk factor of uncontrolled hypertension and smoking in this patient, emergency doctors didn’t consider the diagnosis of SCAD. Regretfully, intravascular ultrasound, which could provide detailed visualization of the arterial wall, was not performed during PCI.

This case shows some clinical implications in differential diagnosis when the AD presenting as AMI. D-dimers are useful to rule in acute AD with an at least moderate discriminatory ability [14]. However, elevated D-dimer levels, which play an important role in predicting prognosis for STEMI [15] and NSTEMI [16], were also seen in patients with AMI. Further investigations were carried out to clarify the value of D-dimer tests in differentiation between AD and AMI [17–19]. Although the cutoff values used in these studies were different, the D-dimer values of patients with AD were higher than those of AMI patients [20]. When a D-dimer level is higher than 0.5 μg/ ml, Li et al. suggested other imaging tests are needed to help doctors rapidly distinguish AD from AMI [17]. The laboratory tests showed the level of D-dimer increased (4.52 mg/L), which raised the suspicion for AD. De Winter ECG pattern drove clinicians to pay more attention to the AMI, and they didn’t order further tests (i.e. TTE, CT angiography) to exclude AD in the setting of elevated D-Dimer. Unfortunately, 7.1% (9/126) of AD exhibited negative D-dimer results [21]. D-dimer is just an assisted biomarker for the diagnosis of AD and lacks sensitivity when used alone. TTE, demonstrating a sensitivity of 77–80% and a specificity of 93–96% for the involvement of the ascending aorta, is recommended as an initial imaging investigation on diagnostic work-up of acute aortic syndrome [22]. Direct TTE signs (i.e. an intimal flap, intramural hematoma and penetrating aortic ulcer) can rapidly identify patients requiring advanced imaging despite low clinical probability [23]. Despite an Aortic Dissection Detection Risk Score of 0 and D-dimer < 0.5 mg/L, indirect signs (i.e. pericardial effusion, aortic regurgitation and dilated aortic root) on ultrasound can offer valuable information that can aid in the diagnosis of AD [24]. Compared to CT and magnetic resonance imaging, TEE can be performed in a critical care area as resuscitative efforts are ongoing. Due to its immediate availability at the patient’s bedside, TTE provides the capability for patients to be sent for immediate surgery instead of a referral for an emergency CT scan of the aorta, which has been shown to improve preoperative mortality [25].Intraoperative ultrasound showed intimal tear in the aortic arch in our patient when unsuccessful attempt to selectively engage the coronary ostium during CAG. Further research is needed to explore that if the point-of-care ultrasound should be performed in all suspected AMI patients prior to PCI to minimize the omission diagnostic rate of AD without delaying the antithrombotic therapy and revascularization.

Cardiologists could find some clues of AD during coronary angiography. I t is important for the operating physician to suspect the diagnosis of AD when unsuccessful attempt to advance the catheter into the coronary ostium. Other important diagnostic auxiliary clues during catheterization are reported, such as obvious pressure changes between the radial artery and the ascending aorta [26] and diametric differences between the aortic root and the ascending aorta following aortography [27].

This patient was received dual antiplatelet therapy and cardiac catheterization after the diagnosis of AMI was made. However, the therapeutic approaches for AMI are absolute contraindications to AD treatment and may result in aortic expansion and rupture for the patient with AD [28]. Fortunately, catastrophic events didn’t occur in this case.

Once acute TAAD is confirmed, surgery is the treatment of choice [22]. However, in cases of extensive myocardial ischemia and hemodynamic instability due to dissection, as is in the present case, what is the appropriate and prompt treatment when the coronary angiography have begun? Maybe the coronary blood flow could be more rapidly restored by stenting in the catheterization laboratory rather than surgical repair of the dissection in the operating room [29, 30].Although stenting to the occluded coronary arterial is controversial, a number of successful cases were reported in the patients with AMI due to AD [31–33]. The aortic replacement surgery couldn’t be performed in our hospital, so PCI was performed immediately on the patient s and gain some time to stabilize cardiac hemodynamic and temporize before surgical intervention could be attempted.

This case report highlights that de Winter ECG pattern is not exclusive to LAD occlusion by atherosclerotic disease but can also be caused by AD which involve LAD. Emergency physicians and cardiologists should rule out AD complicated with AMI when manage a patient with chest pain, high D-dimer levels and de Winter ECG pattern. TTE is an easy approach to evaluate the possibility of AD. When there’s no condition to perform aortic replacement surgery immediately, PCI may be used as a treatment strategy in the case of AD leading to severe LAD occlusion and unstable hemodynamics. To evaluate the feasibility of PCI before aortic replacement surgery in such cases, we need to carry out a series of rigorous clinical trials to collect high level evidence.

## Supplementary Information

**Additional file 1.** Video of coronary angiography.

## Data Availability

The datasets used and/or analyzed during the current study are available from the corresponding author on reasonable request.

## Abbreviations

ECG: Electrocardiograph
LAD: Left anterior descending
STEMI: ST-segment elevation myocardial infarction
PCI: Percutaneous coronary intervention
AD: Aortic dissection
AMI: Acute myocardial infarction
CAG: Coronary angiography
LM: Left main
LCX: Left circumflex
CT: Computed tomography
TAAD: Type A aortic dissection
TTE: Transthoracic echocardiograph
NSTEMI: Non-ST-segment elevation myocardial infarction
SCAD: Spontaneous coronary artery dissection
